# A Practical Treatise on Mechanical Dentistry

**Published:** 1868-12

**Authors:** 


### A PRACTICAL TREATISE ON MECHANICAL DENTISTRY.

BY JOSEPH RICHARDSON, D. D. S., M. D.

Second Edition. Enlarged.

This work, just issued by Lindsay & Blakiston, is the embodiment of the principles involved in Dental mechanism. It has been thoroughly revised, and whatever in the former edition has become obsolete, or been superseded by different and better modes and processes, has been left out, and whatever has been developed, since the first edition, that is valuable to the Dental mechanician, has been introduced in the very happy style of the author.

Without any disparagement, whatever, of works that were written years ago upon this subject, we can truly say that this is the only work that, in this respect, fills the requirements of the Dental student and practitioner. Some new subjects have been introduced, and much of the old matter entirely rewritten, so that the work is fully up to the present time.

No practitioner or student should be without this work, and having the former edition makes it all the more important to have this one.

The truly progressive man will always and immediately have the latest editions of his text and reference books. It is for sale by Robert Clarke & Co., of this city. Price $4 50. T.

